# Diet-Induced Hypercarotenemia in a Patient With Chronic Constipation: A Case Report

**DOI:** 10.7759/cureus.118031

**Published:** 2026-10-16

**Authors:** Jason H Haack, Madison Sullivan, Ashok Tuteja

**Affiliations:** 1 Dermatology, Spencer Fox Eccles School of Medicine at the University of Utah, Salt Lake City, USA; 2 Pediatrics, Brown University, Providence, USA; 3 Gastroenterology, University of Utah and VA Medical Center, Salt Lake City, USA

**Keywords:** carotenoderma, carotenoids, chronic constipation, dietary fiber, hypercarotenemia

## Abstract

Chronic constipation can significantly impair a patient’s quality of life, often leading to physical discomfort and negatively impacting daily activities. The primary treatment for this condition typically includes lifestyle modifications, such as increased dietary fiber intake. This case report highlights a 57-year-old female with chronic constipation who developed hypercarotenemia as a result of physician recommendations to increase her dietary fiber intake. Over her previous eight years of treatment, she significantly increased her dietary fiber intake by eating carrots as a daily snack, resulting in a noticeable yellow-orange discoloration of her palms. Laboratory tests confirmed hypercarotenemia, with elevated carotene levels but normal liver enzyme activity, supporting a diagnosis of diet-induced hypercarotenemia. This case underscores the importance of dietary diversity when recommending increased fiber intake, particularly avoiding excessive reliance on a single carotenoid-rich food. Clinicians should be mindful of signs of hypercarotenemia in patients with excessive intake of carotenoid-rich foods, particularly in cases of unexplained skin discoloration.

## Introduction

Chronic constipation can significantly impair a patient's well-being, and its management can, in uncommon cases, lead to unintended consequences such as diet-induced hypercarotenemia. Symptoms of constipation commonly include hard stools, a feeling of incomplete evacuation, abdominal discomfort, bloating, and distention [1]. These physical symptoms may also lead to increased absenteeism at work or school, increased healthcare utilization, and impairment in daily activities, all resulting in significantly lower mental and physical quality of life [2]. Regardless of the underlying pathology, lifestyle modifications, including increased dietary fiber intake, are often the first recommendations for these patients [3]. Given the substantial impact of chronic constipation, patients are often highly motivated to follow medical advice, sometimes to an extreme. This can pose a hazard to patients if dietary diversity is not emphasized, as fat-soluble vitamins (A, D, E, K) can accumulate to excess with prolonged overexposure, whereas water-soluble vitamins are not stored long-term [4]. Evidence suggests that dietary interventions may be more effective when recommendations are personalized to the individual [5-7]. We report a case of diet-induced hypercarotenemia in a patient with chronic constipation who markedly increased carrot intake after repeated nonspecific counseling to increase dietary fiber.

## Case presentation

A woman in her 50s with a past medical history significant for a severe motor vehicle accident (MVA), anxiety, depression, and chronic constipation presented to the outpatient gastroenterology clinic for continued management of constipation. This constipation developed following her MVA, and she had pursued treatment for over eight years with minimal improvement.

On physical examination, the patient presented with normal vital signs and residual facial deformities related to her prior MVA. Her cardiopulmonary and neurologic examinations were unremarkable. Abdominal examination revealed a soft, non-tender, non-distended abdomen with normoactive bowel sounds. Incidentally, yellow-orange discoloration of the bilateral palms was noted without associated scleral icterus or mucosal discoloration (Figure 1).

**Figure 1:**
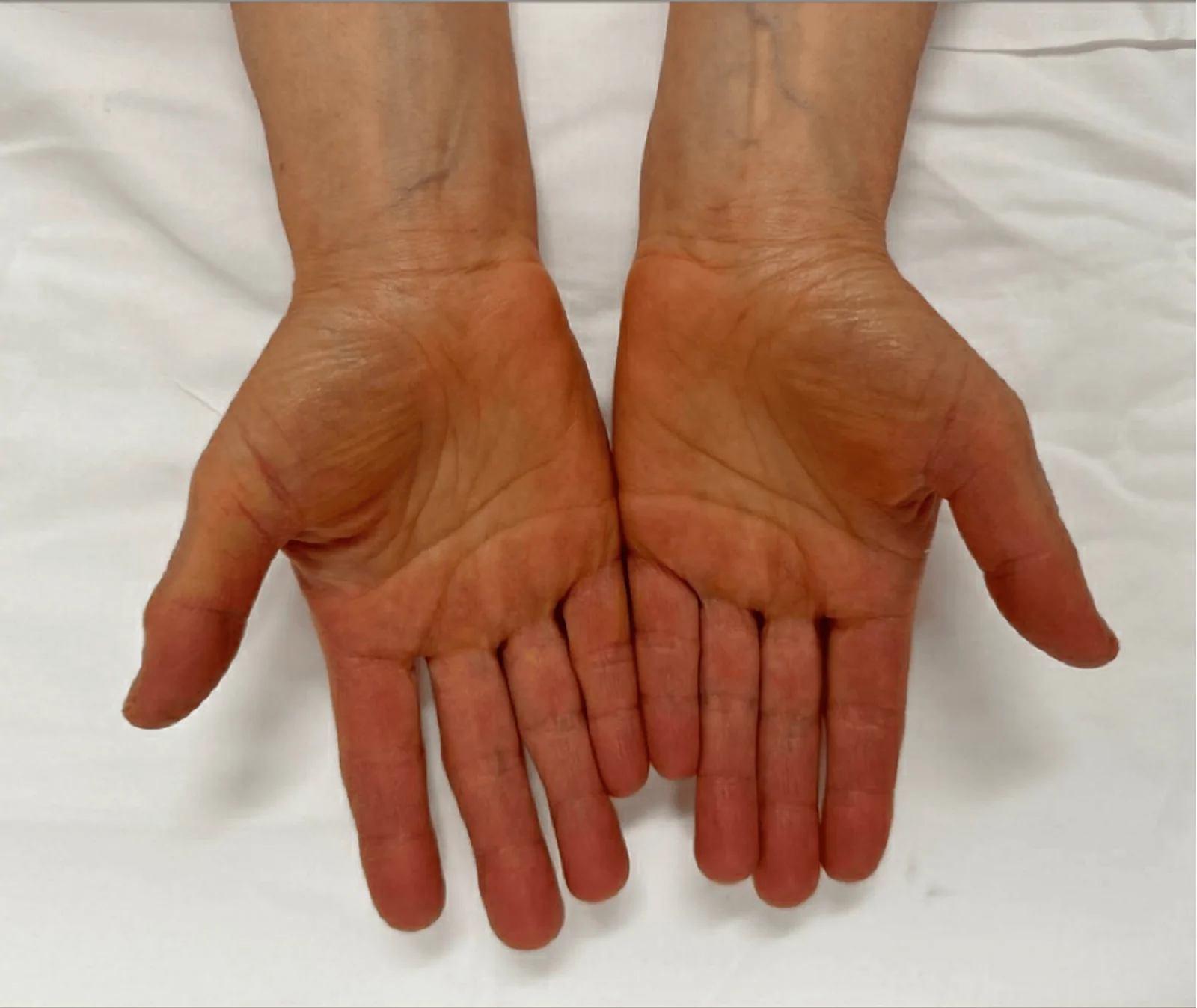
Yellow-orange discoloration of the bilateral palms.

Further questioning revealed that the patient had been repeatedly advised to increase her dietary fiber intake to manage her chronic constipation. Although she initially incorporated a variety of fruits and vegetables into her diet, carrots gradually became her predominant source of dietary fiber. At presentation, she reported consuming at least one cup of shredded carrots with breakfast and carrying baby carrots with her as a daily snack. This had been ongoing for several years, and she associated this change with improvement in her constipation. She reported approximately three bowel movements per week; however, she maintained a sensation of incomplete evacuation and began to experience bloating and excessive gas. She did not initially associate these dietary habits with her skin discoloration.

Given the characteristic palmar discoloration, absence of scleral icterus, and notable dietary history, laboratory evaluation for hypercarotenemia was obtained. Serum carotene concentration was elevated, while liver enzymes remained within normal limits, supporting the diagnosis of diet-induced hypercarotenemia (Table 1). A vitamin A level was not obtained as she did not present with symptoms of hypervitaminosis. 

**Table 1:** Laboratory values at patient presentation and three-month follow-up supporting hypercarotenemia. ALT: alanine aminotransferase; AST: aspartate aminotransferase; eGFR: estimated glomerular filtration rate; H: high; HbA1c: hemoglobin A1c; TSH: thyroid-stimulating hormone

Parameters	Patient Values		Reference Ranges
	Presentation	Follow-Up	
Carotene	539 mcg/dL H	Not collected	(60-200 mcg/dL)
AST	19 unit/L	29 unit/L	(9-40 unit/L)
ALT	15 unit/L	39 unit/L	(0-40 unit/L)
Bilirubin	0.4 mg/dL	0.5 mg/dL	(0.2-1.3 mg/dL)
TSH	Not collected	2.87 uIU/mL	(0.27-4.20 uIU/mL)
HbA1C	Not collected	5.5%	(<5.7%)
eGFR	Not collected	96 mL/min/1.73m²	-

The patient was counseled to reduce her carrot intake and diversify her dietary fiber. At three-month follow-up, her constipation had improved through a combination of medication management and dietary diversity. She was having a daily bowel movement; however, the sensation of incomplete evacuation persisted. As her last colonoscopy was normal, additional evaluation with anorectal manometry was completed, demonstrating dyssynergic defecation, and she was ultimately referred to biofeedback physical therapy for additional management.

Notably, the patient reported reducing her carrot consumption to one to two times per week and that her yellow-orange palmar discoloration had resolved. Repeat testing demonstrated TSH, HbA1C, kidney function, and liver enzymes within normal limits, providing reassurance against secondary hypercarotenemia (Table 1). Because her cutaneous findings resolved following dietary modification and there was no clinical concern for persistent hypercarotenemia, repeat serum carotene measurement was not pursued.

## Discussion

Carotenoderma is the cutaneous manifestation of hypercarotenemia and classically presents as yellow-orange discoloration of the skin, most prominently involving the palms, soles, and nasolabial folds [8,9]. Excess dietary intake of carotenoid-rich foods represents the most common etiology, particularly in infants and young children. However, it may also occur in adults with restrictive dietary habits or chronic consumption of carotenoid-rich foods [10,11]. Secondary causes include hypothyroidism, diabetes mellitus, anorexia nervosa, liver disease, kidney disease, and rare metabolic disorders affecting carotenoid metabolism, such as carotenoid 15,15'-monooxygenase deficiency [12].

Carotenoids are absorbed primarily within the proximal small intestine and subsequently converted to vitamin A within the intestinal mucosa and liver [13]. Because mammals cannot synthesize vitamin A de novo, dietary carotenoids remain an essential nutritional source. Hypercarotenemia results from excessive carotenoid intake or impaired metabolism and is generally considered a benign condition [9]. Over time, excess carotenoids accumulate in sweat, sebum, and the lipid-rich stratum corneum, resulting in the characteristic skin discoloration seen in carotenoderma [14]. The palms and soles are preferentially affected because of their thick stratum corneum and high density of eccrine sweat glands [15]. With time and excessive carotenoid intake, this discoloration can become increasingly generalized and pronounced. Importantly, the sclera and mucous membranes are spared because they lack a stratum corneum, helping distinguish carotenoderma from jaundice [9].

Carrots are a rich source of provitamin A carotenoids and a well-established dietary source of fiber, having been shown to increase fecal bulk, shorten intestinal transit time, and promote bowel regularity [16,17]. Similar cases of diet-induced carotenemia following excessive consumption of carotenoid-rich foods have been described, including a report of marked hypercarotenemia associated with high beta-carotene intake [17,18]. In this case, prolonged adherence to repeated recommendations to increase dietary fiber intake resulted in excessive consumption of a single carotenoid-rich food source. This case highlights the value of specific, individualized fiber counseling over general recommendations to “eat more fiber.”

Laboratory findings in hypercarotenemia typically demonstrate elevated serum carotene concentrations with normal vitamin A levels [17,19]. Management primarily consists of reducing dietary carotenoid intake. Patients can expect the pigmentary changes to normalize with time, though discoloration may persist for weeks to years, depending on the severity and duration of excess intake [9]. When hypercarotenemia occurs secondary to an underlying metabolic or systemic disorder, treatment should additionally focus on the associated condition.

## Conclusions

Dietary fiber intake remains an important component of chronic constipation management. This case illustrates a benign but preventable consequence of disproportionate reliance on a single carotenoid-rich food source: hypercarotenemia and carotenoderma. As this observation is drawn from a single patient, it should not be generalized as a common complication of fiber therapy; rather, it highlights a practical consideration for clinicians - emphasizing dietary diversity, including appropriate intake of fat-soluble vitamins (A, D, E, K), when recommending increased fiber intake. Clinicians should also remain aware that yellow-orange discoloration of the palms and soles with sparing of the sclera may represent benign carotenoderma rather than jaundice.
